# Neurosecretion: what can we learn from chromaffin cells

**DOI:** 10.1007/s00424-017-2051-6

**Published:** 2017-08-11

**Authors:** Erwin Neher

**Affiliations:** Max-Planck-Institut für biophysikalische Chemie, Am Fassberg 11, 37077 Goettingen, Germany

**Keywords:** Chromafffin cell, Calyx of held, Active zone, Vesicle pool, Munc13

## Abstract

Many of the molecular players in the stimulus-secretion chain are similarly active in neurosecretion and catecholamine release. Therefore, studying chromaffin cells uncovered many details of the processes of docking, priming, and exocytosis of vesicles. However, morphological specializations at synapses, called active zones (AZs), confer extra speed of response and another layer of control to the fast release of vesicles by action potentials. Work at the Calyx of Held, a glutamatergic nerve terminal, has shown that in addition to such rapidly released vesicles, there is a pool of “Slow Vesicles,” which are held to be perfectly release-competent, but lack a final step of tight interaction with the AZ. It is argued here that such “Slow Vesicles” have many properties in common with chromaffin granules. The added complexity in the AZ-dependent regulation of “Fast Vesicles” can lead to misinterpretation of data on neurosecretion. Therefore, the study of Slow Vesicles and of chromaffin granules may provide a clearer picture of the early steps in the highly regulated process of neurosecretion.

## Introduction

It was a streak of luck that a nearby laboratory at our institute was working on chromaffin cells at the right time. We had just discovered that currents and voltages could be recorded from whole cells by breaking a patch after obtaining a tight seal (the whole-cell variety of the patch clamp technique [14]) and pondered whether this type of getting access to the interior of cells might be more gentle and better tolerable for small cells, as compared to the classical recording method of pushing sharp electrodes through the plasma membrane. So we were looking for small cells, for which electrophysiological recording had been difficult so far. And here was Elizabeth Fenwick, studying the biochemistry of catecholamine release in Victor Whittaker’s department at our institute. She was dissociating adrenal glands a few times per week, preparing suspensions of millions of chromaffin cells—and we needed only few hundreds to test whether “whole-cell recording” would work on these cells. Had there been an immunology laboratory next door, we might have started working on lymphocytes.

Our interest in chromaffin cells was mainly methodological. However, after we realized that these cells had Na^+^-channels, Ca^2+^-channels and BK-channels [13, 20], we were happy to use them as testbed for ion channels. Still, the real function of chromaffin cells as sources of catecholamines and as key players in endocrinology was not of much relevance in our agenda. This changed, when Antonio Garcia appeared on our horizon. I met Antonio first during the second International Symposium on Chromaffin Cell Biology held in 1984 in Colmar—part of a conference series initiated by Antonio at Ibiza in 1982. The talks given convinced me that the chromaffin cell had an important role of its own and understanding of its channels, its Ca^2+^ signals, and eventually its catecholamine release, might constitute a major contribution to endocrinology. So, I was quite happy when Antonio invited me to come to Madrid and to spend a short sabbatical in his laboratory. My stay in Madrid resulted in the introduction of patch clamp into Antonio’s lab and, in the follow-up, in a number of joint publications both with Antonio [3, 18, 32] and with members of his laboratory [2, 26, 37]. In this work, which extended between 1990 and 2005, we addressed a number of chromaffin-cell-specific problems, but still my interest was focused on issues like [Ca^2+^]-signaling and the secretory process per se.

When part of my laboratory switched to study synaptic neurotransmitter release, we initially took chromaffin cells as a model for nerve terminals. This proved useful for a number of aspects - in particular regarding the role of some synaptic proteins in exocytosis [27]. However, the comparison of two secretory systems also revealed a number of fundamental differences between catecholamine secretion and neurotransmitter release. These are mainly rooted in the fact that nerve terminals contain Active Zones (AZ), highly structured specializations, where Ca^2+^-channels are found in a defined and tight spatial relationship to release-ready vesicles and their Ca^2+^-sensors. I summarized my view on these differences in a short review in Pflügers Archiv [22]. Here, I would like to draw the attention to some recent literature findings on neurotransmitter release, which I think highlight what we can learn from studies on chromaffin cells.

## Some differences between neurosecretion and catecholamine release

While many of the molecular players are the same in both secretory systems [35], some functional differences are quite pronounced. As detailed in [22], the major differences are:

- The pool of release-ready vesicles, which leads to an exocytotic burst upon strong stimulation, is dynamic in chromaffin cells, depending on the basal level of free intracellular Ca^2+^-concentration ([Ca^2+^]) and other signaling pathways. In the Calyx of Held, a glutamatergic nerve terminal in the central nervous system, the number of release-ready vesicles is changing much less in response to such modulatory influences. However, other types of synapses in other brain areas do show pronounced variation, depending on second messengers and preceding stimulation (see below).
- In both secretory systems, the population of release-ready vesicles is not homogenous, but can be subdivided into pools of different release “willingness” or speed of release. However, the mechanisms, which underlie such differences, are not the same in the two systems. In chromaffin cells, recent findings point towards a rapid step of priming, which “slow” vesicles have to undergo, before they can respond to a stimulus [19, 35, 39]. In contrast, the main subdivision of release-ready vesicles between fast ones and slow ones at the Calyx of Held was attributed to the question of whether vesicles are tightly linked to the AZ and, therefore, located nearby Ca^2+^ channels, or else more remote [7, 23, 34]. This distinction is important, since I will argue below, that destroying specific interactions between vesicles and AZ-proteins will make neurotransmitter release more similar to catecholamine release. Recent work [30] showed that at the nerve terminal “fast” vesicles should be subdivided into so-called “normally primed” and “superprimed” ones, the latter ones displaying extra-high release probability. This may be related to the finding in insulin-secreting cells of a “highly Ca^2+^-sensitive pool of granules,” regulated by glucose and protein kinase C [38].
- The role of active zone proteins, such as Rim and Rim-binding proteins: Although a pool of vesicles, which seems to sense higher than average [Ca^2+^] during depolarization and Ca^2+^ influx was identified in adrenal chromaffin cells [33] the spatial organization determining distances between Ca^2+^ channels and docked granules does not seem to be very tight. In contrast, specialized proteins of the nerve terminal, such as Rim and Rim Binding proteins establish a scaffold for both Ca^2+^ channels and release-ready granules, which enable tight and well-defined coupling [12, 15, 36]. Also, various types of synapses use unique morphological specializations, such as ribbons, T-bars, and linear arrays for fine-tuning the steps in the stimulus-secretion sequence [6, 21]. I am not aware of reports on similarly dedicated structures in chromaffin cells.

## The dynamic “Ready-Releasable Pool”

The “classical” glutamatergic synapses in the mammalian central nervous system, such as the CA3-CA1 synapse of the hippocampus or the Calyx of Held synapse harbor at rest a stable “readily releasable pool” (RRP) of vesicles, which allows them to generate robust postsynaptic currents upon stimulation [1, 11, 23]. However, many other types of synapses display very small EPSCs after periods of rest and undergo strong, slowly developing facilitation during repetitive stimulation [8, 25, 40]. They remain potentiated over tens of seconds, but lose most of their RRP subsequently. This behavior—although generally interpreted in terms of facilitation—is reminiscent of findings in chromaffin cells, where the exocytotic burst, the equivalent of the RRP, is strongly dependent on [Ca^2+^] preceding stimulation and also shows “depriming,” when basal [Ca^2+^] is reduced after periods of elevation [10, 28]. Both types of synapses are found at the crayfish neuromuscular junction, where they are called “phasic” (those showing a large initial response, which, however, decays during high-frequency stimulation due to pool depletion) and “tonic” (the facilitating ones). A study of these synapses [25] explained differences between the two types by assigning a stable RRP to the phasic synapses, while invoking depriming at low [Ca^2+^] to explain the decrease of the RRP during periods of rest in the tonic synapses.

Molecular mechanisms, which may be responsible for the instability of the RRP and the difference between phasic synapses on the one hand and tonic synapses and chromaffin cells on the other hand have recently been put forward. In a study on the SM-proteins Munc13 and Munc18, He et al. [16] found that synapses of hippocampal neurons were able to maintain a stable RRP at rest only if they expressed the wild type isoforms Munc13-1 and Munc18-1. In synapses from knockout animals, in which either one of these proteins was rescued by Munc13-2 or Munc18-2, the RRP was much reduced and displayed activity-dependent potentiation and depriming, as is the rule in chromaffin cells and in tonic synapses. Intriguingly, Munc13-2 was found in another recent study to be the main isoform of this protein in mouse chromaffin cells [19]. Taking these results together, one can conclude that Munc13-1 and Munc18-1 are required for a stable RRP and that studies on chromaffin cells [19, 39] may lead to an understanding of why neurotransmitter release in tonic synapses is different from that in phasic ones.

Two more recent findings shed light on the mechanisms, which regulate stability of the RRP. First, a very strong phenotype, similar to the one described for the Munc13-2/Munc18-2 synapses was found for synapses lacking the protein CAPS-1 [17]. This finding adds CAPS-1 to the list of proteins needed for a stable RRP. Secondly, He et al. [16] also showed that the instable RRP in synapses expressing Munc13-2 or Munc18-2 can be stabilized by blocking the protein N-ethylmaleimide sensitive factor (NSF). Thus, Munc13-1 and Munc18-1 seem to be able to protect partially assembled SNARE-complexes against the attack of NSF, which is well known to disassemble cisSNARE-complexes after vesicle fusion. An intriguing, and still puzzling finding is that blocking NSF does not stabilize the RRP in synapses lacking CAPS [16]. Maybe studies on chromaffin cells which already identified major differences in the priming action of CAPS and Munc13 proteins [31] will shed light on the specific roles of the proteins involved.

## Destroying the active zone (AZ)

If, as argued above, the presence of the AZ were responsible for many of the special properties of nerve terminals, one would predict that eliminating some of the major components of the AZ might render neuronal secretion more similar to that observed in chromaffin cells.

Experiments, describing such manipulations were recently published—although not discussed in their relation to chromaffin cells. Wang et al. [36] found that simultaneous conditional knockdown of ELKS and Rim proteins resulted in the loss or reduction of most other active zone proteins, including a strong reduction of Munc13-1. This way, the active zone was actually abolished, which led to a reduction in vesicle docking and release probability for single action potential stimulation. However, surprisingly, a pool of vesicles, releasable by either high-frequency stimulation or else by hypertonic solution, remained. Also, spontaneous release was only reduced by about 50%. During high-frequency stimulation, strong facilitation was observed. This was interpreted as the consequence of a Ca^2+^ buildup during stimulation, such that action potentials later in the train were able to release vesicles, whereas single action potentials were less effective than those in wild-type synapses due to less tight coupling between Ca^2+^ channels and vesicles. Less tight coupling, low levels of Munc13-1, and release in the absence of a specialized active zone are features, which also characterize catecholamine secretion in chromaffin cells. It would be interesting to explore, whether the observed facilitation and tonic release during high-frequency stimulation, as observed after destruction of the AZ also includes a dynamic up-regulation of priming. Such an additional effect might be due to Munc13-2, which, although expressed at low levels, may take over control of SNARE-complex assembly, when Munc13-1 levels are reduced.

## The sequence of steps in the stimulus-secretion cascade

In recent literature, the sequence of steps leading to exocytosis is often described as “vesicle tethering,” followed by docking, priming, and Ca^2+^-dependent exocytosis. The priming step is held to start with the activation of Munc13 by Rim, followed by Munc18- and Munc13-assisted SNARE complex formation [5, 29]). The Munc13-Rim interaction is considered as the starting point of priming due to the finding that Munc13 can exist in the form of a self-inhibited homodimer, which by interaction with Rim needs to be converted into an active Rim/Munc13 heterodimer [9, 12]. This sequence of events, however, is incompatible with the ideas discussed here:

- No major role in vesicle priming is attributed to the Rim-Munc13 interaction in chromaffin cells [4].
- Release and pool size of about 50% of wild type persists in neurons after elimination of both Rim1 and Rim2 [36].
- Many of the findings regarding the pool of slow vesicles at the Calyx of Held can be explained by the assumption that they represent release-competent vesicles with a fully developed release apparatus before the interaction with Rim. In this view, a late step of interaction of the vesicle with the active zone (e.g., the formation of the tripartite Rim-Rab3-Munc13 complex (12)) converts slow vesicles into fast ones by “positional priming,” i.e., by bringing them closer to Ca^2+^ channels [22, 24].

Taken together, it seems reasonable to draw the dividing line between catecholamine granules and slow synaptic vesicles on the one hand and “positionally primed” vesicles at the active zone on the other hand. The study of the latter is certainly attractive, since the morphological specializations confer high speed and an additional layer of regulatory mechanisms to neurotransmitter release, which may be important for the understanding of synaptic plasticity. The added complexity of the neuronal system, however, also adds to the difficulty in interpreting experimental findings. Studies of catecholamine secretion may, therefore, continue to teach us a lot about the basic mechanisms of vesicle tethering, docking, and the control of exocytosis.
